# Humanization of AI-generated abstracts in oral radiology: Role of chatbots and dedicated platforms

**DOI:** 10.1590/1807-3107bor-2026.vol40.067

**Published:** 2026-08-21

**Authors:** Caio Alencar-Palha, Thaís Ocampo, Thaísa Pinheiro Silva, Deborah Queiroz Freitas, Frederico Sampaio Neves, Matheus Lima Oliveira

**Affiliations:** (a) Universidade Estadual de Campinas – Unicamp, Piracicaba Dental School, Department of Oral Diagnosis, Piracicaba, SP, Brazil; (b) Universidade Federal da Bahia – UFBA, School of Dentistry, Department of Propaedeutics and Integrated Clinic, Salvador, BA, Brazil.

**Keywords:** Artificial Intelligence, Abstracts, Large Language Models

## Abstract

The rise of generative artificial intelligence (AI) in scientific writing has prompted the development of platforms designed to "humanize" AI-generated text by rephrasing content to reduce its detectability as machine-generated. This study aimed to evaluate the performance of chatbots and dedicated platforms in humanizing AI-generated abstracts. Thirty high-impact scientific articles published in dentomaxillofacial radiology journals were selected. The Methodology and Results sections of each abstract were used to prompt two chatbots—ChatGPT and Gemini—to generate complete abstracts. These AI-generated abstracts were then humanized using the same chatbots along with two dedicated platforms: HumanizeAI and StealthWriter. The percentage of AI-generated content was determined for all abstracts (n = 330), including the original human-written abstracts, using GPTZero. AI detection rates were compared using two-way analysis of variance followed by Tukey's post hoc test (α = 0.05). The results showed that original abstracts and AI-generated abstracts humanized by HumanizeAI and StealthWriter exhibited significantly lower GPTZero detection rates (*p* < 0.05), whereas abstracts generated or humanized by chatbots showed significantly higher detection rates (*p* < 0.05). In conclusion, chatbot-generated scientific abstracts humanized by dedicated platforms exhibit low AI detection rates, whereas those humanized by chatbots exhibit considerably higher detection levels. Educators must prepare students with critical appraisal skills to identify AI-generated content and uphold academic integrity, ensuring that AI serves as a complementary tool rather than a means of misrepresentation.

## Introduction

Artificial intelligence (AI) has played an increasingly prominent role across various fields, including academia.^1^ Tools such as ChatGPT, developed by OpenAI, and Gemini, developed by Google, are examples of large language models (LLMs), which are AI systems designed to generate human-like text. These LLMs can generate high-quality text automatically, sparking a growing debate over the use of AI in producing abstracts, articles, and other forms of scientific content.^2-5^ However, LLMs are also limited by their dependence on the quality of training data, the possibility of producing factually incorrect or biased information, and their limited ability to understand context, because they interpret meaning primarily through pattern recognition rather than true comprehension.^6^ These factors must be considered when assessing their impact and risks in scientific communication.

In recent years, academic authors have increasingly relied on traditional language-editing and translation tools—such as Grammarly, DeepL Translator, and others—to improve the clarity, style, and linguistic accuracy of scientific writing.^5^ While these tools are designed to support human-authored text, the introduction of LLMs has made it possible to generate entire scientific abstracts or manuscripts automatically. Unlike traditional tools, modern LLMs can produce content with minimal human intervention. These LLM-based chatbots offer several advantages in academic contexts, particularly for individuals whose native language is not English.^7^ Their ability to generate text rapidly allows researchers to save time, especially during the early stages of writing, such as drafting abstracts and articles.^8^ Additionally, they can help translate ideas clearly and accurately, enabling researchers from diverse linguistic backgrounds to communicate more efficiently and in accordance with international academic standards.

Despite the advantages offered by these chatbots, several concerns are associated with their use in academic contexts.^9,10^ One of the main concerns is the potential for excessive dependence on these tools, which could affect researchers’ ability to independently develop writing and critical thinking skills.^11^ Another risk involves the accuracy of the information provided, because ChatGPT may generate imprecise or outdated responses.^12,13^ Moreover, ethical implications must also be considered, including the issue of authorship of AI-generated texts and the potential for plagiarism, which raise concerns about transparency and academic integrity.^12^

Scientific abstracts play a crucial role in academic communication. As one of the first points of contact readers have with a paper, they provide a concise and informative synthesis of the study's objectives, methods, results, and conclusions. In many cases, abstracts can influence a researcher's decision to consult the full text, highlighting their importance in both academic communication and knowledge dissemination. Human judgment in detecting AI-generated texts is often limited.^14^ Conversely, online AI detectors have shown promising performance in identifying texts generated by tools such as ChatGPT.^2,15^ These tools analyze linguistic patterns, sentence structures, and stylistic features typical of language models. They are designed to detect inconsistencies that may indicate the use of AI, such as a lack of coherence in parts of the text or the repetition of similar sentence structures.

The "humanization" of AI-generated texts, a recent and still underexplored concept in the literature, represents the use of tools designed to reduce the detectability of AI-generated content. In this study, the term is used solely to describe the function of the dedicated platforms examined and does not imply endorsement or normalization of academic dishonesty. Although this technique may improve the apparent quality of generated content, it also raises serious ethical concerns. This could undermine the reliability of AI detectors that have previously demonstrated promising performance.^2,15^ Consequently, weakening the capacity of these detectors may increase the risk of compromising academic integrity, authorship attribution, and transparency in scientific publishing, particularly when such tools are used without appropriate oversight. Therefore, this study aimed to evaluate the performance of chatbots and dedicated platforms in humanizing AI-generated abstracts.

## Methods

This study was exempt from review by the Human and Animal Research Ethics Committee because it consisted of a retrospective, experimental, and comparative analysis that used previously published scientific articles subjected to the AI-based experimental conditions described herein.

### Selection and generation of scientific abstracts

A total of 30 articles were collected from peer-reviewed dentomaxillofacial radiology journals. An a priori sample-size calculation was not feasible because no previous studies with a comparable methodological design were available to estimate expected values. Therefore, an initial sample of 30 articles was selected. A post hoc power analysis confirmed that this sample size was sufficient for the analyses performed, as described in the AI-generated content detection section. As an inclusion criterion, only articles published before November 2022—when ChatGPT (OpenAI, San Francisco, USA) became available worldwide and Gemini (Google, California, USA) had not yet been released—were included to eliminate potential bias arising from the possibility that these chatbots had been used in any part of the manuscripts. The publication dates of these articles ranged from August 2020 (earliest) to November 2022 (most recent). The Methodology and Results sections of all articles were collected and used as the basis for generating chatbot abstracts.

Two chatbots—ChatGPT-4o Mini and Gemini—were used to generate the abstracts using the following prompt: "Can you generate an abstract with objectives, methods, results, and conclusion, up to 250 words, based on the information below?" This prompt was followed by the Methodology and Results sections from each article. These chatbots were selected because, at the time of data collection, their interfaces allowed the full Methods and Results sections to be entered within a single prompt, which was not technically feasible with other tools that imposed more restrictive character limits per request. Each original abstract was used to generate two chatbot-produced abstracts, resulting in 90 abstracts at this stage (30 original, 30 generated by ChatGPT-4o Mini, and 30 generated by Gemini).

### Humanization of chatbot-generated abstracts

The chatbot-generated abstracts (n = 60) were subjected to a humanization process using four different approaches: two chatbots—ChatGPT-4o Mini and Gemini—and two dedicated humanization platforms, HumanizeAI (available at https://www.humanizeai.io/) and StealthWriter (available at https://stealthwriter.ai).

ChatGPT-4o Mini and Gemini humanized the abstracts using the following prompt: "Can you rewrite this AI-generated scientific abstract into natural human writing while maintaining the 250-word limit?" followed by each chatbot-generated abstract. To ensure that this approach reflected real-world behavior, no prompt engineering or complex instructions were used; instead, the design aimed to simulate common everyday usage by non-expert users and to assess detection rates under typical user conditions.

HumanizeAI and StealthWriter allow users only to paste or upload the text to be humanized, without options for prompt customization or detailed instructions. The abstracts were uploaded individually to these platforms and processed for humanization. For StealthWriter, a humanization level of 10—the maximum available level—was selected to ensure the most comprehensive text modification available within the platform's capabilities. Unlike ChatGPT and Gemini, which allow customizable prompts, StealthWriter allows users to adjust the humanization intensity only through a numerical scale.

The resulting 330 abstracts—30 original abstracts, 60 AI-generated abstracts (30 from ChatGPT-4o Mini and 30 from Gemini), and 240 humanized chatbot-generated abstracts (60 chatbot-generated abstracts processed using four different humanization approaches)—were compiled independently into PDF files. This distribution is illustrated in Figure.

**Figure:**
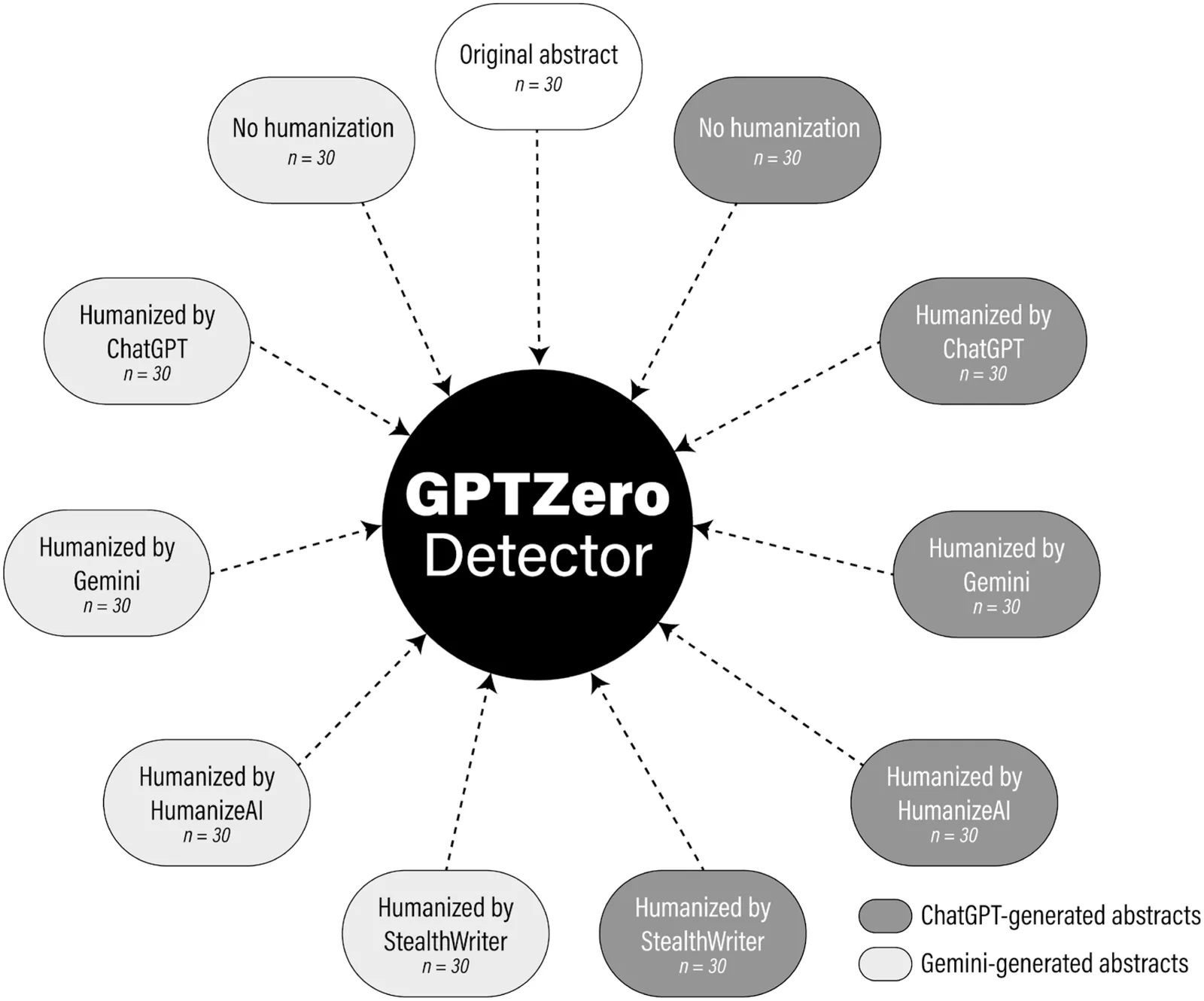
Overview of groups included in the AI detection analysis by GPTZero. Each box indicates a group of 30 abstracts: original, non-humanized (AI-generated by ChatGPT – dark gray – and Gemini – light gray), and humanized by four different platforms. All abstracts were processed by GPTZero to assess the likelihood of AI authorship, allowing comparison of different text-generation and humanization strategies.

### AI-generated content detection

All 330 abstracts were analyzed for AI-generated content using GPTZero, a freely available detector accessible at https://gptzero.me. GPTZero assigns a percentage indicating the likelihood that a given text was generated by AI, with lower values reflecting a reduced probability of AI involvement. The AI-generated content percentages for all 330 abstracts were compared using two-way analysis of variance (ANOVA), followed by Tukey's post hoc test at a significance level of 5% (α = 0.05). The two factors analyzed were the abstract generator and the humanization process. Original articles served as controls for comparing human-authored texts with chatbot-generated texts. A post hoc power analysis based on the differences among the groups, their standard deviations, and the number of observations per group indicated that the sample size ensured a statistical power of 90%. The null hypothesis was that no difference existed in the detection rate of AI-generated content among abstract generators and humanization approaches.

## Results

Table presents the mean values and standard deviations of AI-generated content detection rates according to the abstract generator and humanization approach. Original abstracts had a mean AI detection rate of 10.57%, whereas the non-humanized abstracts generated by ChatGPT and Gemini exhibited significantly higher detection rates (*p* < 0.05). Specifically, ChatGPT-generated abstracts had an AI detection rate of 85.70%, whereas those generated by Gemini had a detection rate of 71.07%. Additionally, non-humanized abstracts generated by ChatGPT had a significantly higher AI detection rate than those generated by Gemini (p < 0.05).

**Table:** Mean (SD) of the detection rates (%) of AI-generated content according to the abstract type and humanization process.

Humanization approach	Abstract generator	
	ChatGPT	Gemini
ChatGPT	86.87 (18.11) b*	94.83 (11.70) c*
Gemini	94.47 (17.52) b*	87.97 (25.81) c*
Humanize AI	6.67 (6.59) a	12.70 (18.42) a
Stealth writer	2.80 (1.16) a	3.10 (2.52) a
No humanization	85.70 (24.27) b*	71.07 (31.57) b*,**
Original abstract	10.57 (20.25)	

Different lowercase letters indicate significant differences within the same column.

* Significantly different from the original abstracts.

** Significantly different from ChatGPT-generated abstracts. (p < 0.0001, two-way ANOVA followed by Tukey post hoc test; statistical power = 90%).

The AI detection rates of humanized ChatGPT- and Gemini-generated abstracts did not differ from each other, regardless of the humanization approach (*p* > 0.05). The two dedicated humanization platforms, HumanizeAI and StealthWriter, exhibited significantly lower AI detection rates than those produced by the chatbots ChatGPT and Gemini for abstract humanization (p < 0.05). Their rates also did not differ from those of the original abstracts (p > 0.05). Notably, only Gemini-generated abstracts showed significantly higher AI detection rates after humanization using ChatGPT or Gemini than before humanization (p < 0.05).

## Discussion

The present study demonstrated that chatbot-generated abstracts, when processed using dedicated humanization platforms, exhibited significantly reduced AI detection rates according to GPTZero. AI detectors have been widely used to identify AI-generated content.^2,15,16^ However, the humanization platforms HumanizeAI and StealthWriter exhibited AI detection rates that did not differ significantly from those of the original human-written abstracts. This finding raises concerns regarding the reliability of AI detectors as the sole tool for validating text authorship and reinforces the need for more robust approaches to differentiate genuinely human-authored texts from artificially modified content. Therefore, implementing clear guidelines and explicitly declaring AI use in scientific publications may be essential for maintaining research credibility and ensuring author accountability for published content. The selected timeframe (2020–2022) was intended to capture abstracts representative of modern academic writing practices, when the widespread use of digital editing tools had already become common. This approach ensured relevance to contemporary scientific communication.

At the time this manuscript was written, no studies in the scientific literature had examined the use of dedicated humanization platforms, such as HumanizeAI and StealthWriter, as strategies to minimize AI detection. Strategies such as introducing minor grammatical errors, using paraphrasing platforms, employing prompts that increase text perplexity and burstiness, and substituting Latin letters with visually similar Cyrillic characters have previously demonstrated the early vulnerability of AI detectors.^17^ However, because of continuous technological evolution, many AI-generated content detectors have improved their performance and shown promising results. GPTZero was selected for the present study partly because of its improved performance and also because it is freely available and easily accessible.^16,18^

The present study investigated the "humanization" of fully AI-generated content—abstracts created entirely by LLMs—using dedicated humanization platforms and chatbots. This approach differs from traditional writing aids intended for language enhancement because the focus here was to assess how these humanization processes affect AI detection in content not originally authored by humans.

Paradoxically, in this study, the original scientific abstracts exhibited an average AI detection score of 10.27%, with some abstracts reaching values between 11% and 20%. This finding may be explained by certain linguistic features used by AI detectors to assess authorship, such as perplexity and burstiness.^19^ Perplexity refers to the predictability of the next word in a sequence, whereas burstiness represents the variation in perplexity throughout the text. AI-generated texts tend to follow consistent patterns in word selection, resulting in lower burstiness. However, the calculation of perplexity and burstiness may sometimes be biased when applied to human writing with limited English proficiency.^20^

The propensity of AI detection tools to produce false positives—classifying human-written abstracts as AI-generated—must also be considered. These findings reinforce the need for cautious interpretation of AI detection outputs and highlight the importance of continued improvement and validation of these tools to ensure their reliability in academic assessment contexts. This may explain why the original abstracts in our study exhibited higher AI detection scores than most abstracts humanized using the dedicated humanization platforms HumanizeAI and StealthWriter, although the difference was not statistically significant.

Artificial intelligence has significantly impacted the academic landscape through LLMs, which serve as the underlying architecture of these chatbots. These effects include improvements in writing by non-native English speakers, personalization of educational experiences through virtual environments, and time savings for researchers resulting from AI's ability to synthesize information and manage data efficiently.^1,21^

However, indiscriminate use without proper supervision still raises concerns regarding reliability and truthfulness.^22^ Beyond what has previously been described as "hallucinations" or "confabulations," caution remains necessary when using LLMs because they lack the capacity to verify the accuracy of the information they generate.^22^ These ethical concerns are fundamental to maintaining reliability and accountability in academic research.

Despite the widespread adoption, accessibility, and previously demonstrated satisfactory performance of GPTZero as an AI-generated content detector, different detectors may yield outcomes that differ from those reported herein. Another limitation is that AI detectors can incorrectly classify some human-written abstracts as AI-generated. In our dataset, original abstracts showed a median detection probability of 10.57%. This finding demonstrates the risk of false positives and underscores the need for caution when interpreting AI detection results. However, the present methodological design addresses these limitations by using a single detector to test four humanization approaches.

Additionally, the effectiveness of humanization may vary depending on the abstract generator, highlighting the need for future research to assess the performance of textual humanization under different experimental conditions. Further investigations should also explore the influence of authors’ native language on detection rates and examine how different academic writing styles impact detector accuracy. Advancing these research areas could contribute to the development of more robust and transparent methods for identifying AI-generated texts in scientific literature.

The use of a single AI-detection system—which may not fully capture variability in performance among different detectors and algorithms—should be considered when interpreting the findings. Although previous studies^2,3,15^ have reported reasonably high accuracy for AI-detection tools when distinguishing fully human-written from fully AI-generated texts, these systems are not error-free, and false positives and false negatives remain a concern.

Given these findings, promoting transparency regarding the use of generative AI tools and humanization platforms is essential in both dental education and scientific publishing. Educational strategies should be reevaluated, placing greater emphasis on developing critical thinking, ethical reasoning, and practical competence rather than solely on written assignments that may be susceptible to AI assistance. Alternative forms of assessment, such as oral examinations, peer discussions, and reflective analyses, may support deeper understanding and academic integrity. Ultimately, preparing students and researchers to use these technologies responsibly can help harness their educational potential while safeguarding academic and scientific standards.

## Conclusion

Chatbot-generated scientific abstracts humanized by dedicated platforms exhibit low AI detection rates, whereas those humanized by chatbots exhibit considerably higher detection levels. These findings underscore the importance of developing rigorous guidelines for the ethical use of AI in academic writing and research. Rather than endorsing uncritical use, stakeholders are encouraged to develop transparent disclosure policies, establish ethical frameworks for AI assistance, and implement diversified assessment methods to safeguard academic integrity.

## Data Availability

The authors declare that all data generated or analyzed during this study are included in this published article.
